# Deep Learning for Automated Tic Detection and Prediction in Tourette Syndrome Using Electromyography and Video

**DOI:** 10.21203/rs.3.rs-10832475/v1

**Published:** 2026-09-20

**Authors:** Grace Lowor, Jiaqing Zhang, Julieth Gomez, Jackson Cagle, Christopher R. Butson, Kelly Foote, Michael S. Okun, Parisa Rashidi, Aysegul Gunduz

**Affiliations:** 1 Norman Fixel Institute for Neurological Diseases, University of Florida, Gainesville, FL 32608, USA; 2 Department of Biomedical Engineering, University of Florida, Gainesville, FL 32611, USA; 3 Department of Electrical and Computer Engineering, University of Florida, Gainesville, FL 32611, USA; 4 Department of Neurology, University of Florida, Gainesville, FL 32608, USA; 5 Department of Neurosurgery, University of Florida, Gainesville, FL 32608, USA

**Keywords:** Tourette Syndrome, tics, electromyography, video, neural networks

## Abstract

Tourette syndrome is characterized by involuntary motor and vocal tics, which are challenging to monitor objectively. This study evaluated convolutional neural networks (CNNs) and Transformer models on EMG and video data (‘Rest’, ‘Tic’, and ‘Voluntary Movement’) for detection and prediction. We hypothesized that EMG windows <5s would better capture tics and improve detection and prediction accuracy. 685 EMG recordings (n=16 participants) were each segmented into 1-, 5-, and 10-second windows. Each model was trained on clinical EMG and validated on held-out data, using corresponding expert-annotated video labels as ground truth. We evaluated model performance using the area under the receiver operating characteristic curve (AUROC) with 95% confidence. Detection performance exceeded prediction across models. The temporal CNN achieved the highest detection performance (mean AUROC 0.66±0.05), with optimal results in 5s windows (AUROC up to 0.82 for tic detection). In contrast, tic prediction performance was lower (best 1s-AUROC 0.66 (0.65-0.68)). Contrary to our hypothesis, longer temporal windows (≥5s) improved tic detection, suggesting that tic-related EMG patterns benefit from extended temporal context, whereas prediction relied on short-term, 1-second signal dynamics. These findings help define a benchmark and suggest that future progress will depend on multimodal integration, patient-specific adjustments, and advanced temporal modeling.

## Introduction

Tourette Syndrome (TS) is a complex neurodevelopmental disorder characterized by involuntary, repetitive motor and vocal tics that typically begin in childhood and frequently persist into adulthood ^1–4^. Affecting nearly 1% of the U.S. population, TS frequently co-occurs with psychiatric conditions such as attention-deficit hyperactivity disorder (ADHD), obsessive–compulsive disorder (OCD), anxiety, and depression, all of which can compound the psychosocial burden of the disorder ^5,6^. The fluctuating nature of tics, their variable intensity and frequency, and their susceptibility to suppression and environmental context present unique challenges for accurate clinical assessment of tics. Evaluating treatment effectiveness in TS is crucial; however, current assessment methods rely predominantly on subjective rating scales or brief observational protocols, and these may fail to capture the full scope of tic expression over time.

Conventional tic assessment tools can be categorized into two types: patient recollection-based and observation-based scales. Recollection-based tools such as the Yale Global Tic Severity Scale (YGTSS) ^7^ have been widely used in both clinical and research settings due to robust psychometric properties. However, these measurements are susceptible to patient recall bias and rely heavily on patient or caregiver reports of tic frequency, severity, and associated impairment over the preceding week ^7^. Observation-based approaches, such as the modified Rush Video-Based Tic Rating Scale (MRVTRS), have attempted to overcome these limitations by scoring tic behavior from short video segments recorded in the clinic ^8^. While this method has improved objectivity, it remains constrained by the brief observation window (5 minutes), the requirement for expert scorers, and the variability and suppressibility of tics during clinical visits ^9–11^.

To address these limitations, there has been a growing interest in automated, objective methods for tic detection that leverage multimodal data and machine learning. Video analysis can capture a wide range of body and facial movements, while electromyography (EMG) can enable high-resolution measurement of muscle activity, including subtle tics that may escape visual detection ^12–14^. However, each modality alone has limitations. Video is sensitive to environmental conditions and occlusions, and EMG cannot detect vocal tics ^15,16^. This study builds on our previous work comparing video- and EMG-based tic detection ^15,16^ by introducing an artificial intelligence (AI) framework to overcome methodological limitations of earlier approaches and enable more robust multimodal motor tic detection for clinical and research applications. Our previous framework further demonstrated the value of integrating time-series data and visual information ^16,17^. Combining the strengths of EMG and video modalities, this study aimed to enhance the performance of EMG-based neural networks for tic classification and prediction across different observation windows. We hypothesized that shorter EMG observation windows (<5 seconds) would improve model performance for tic detection, given the brief, burst-like nature of tics ^18^. The objective of this study was to establish a more reliable, scalable, and objective framework for tic detection and prediction. The data from this study were designed to be used in future systems to implement objective clinical measures and to improve clinical monitoring, treatment evaluation, and creation of more reliable closed-loop neuromodulation systems.

## Results

### Detection Task

We evaluated both the CNN models and the transformer for detecting “Rest”, “Tic”, and “Move” within the defined temporal model windows, using AUROC with a 95% confidence interval (CI) (Table 2). The tCNN outperformed the others in detecting all classes with an AUROC (mean ± std) of 0.66 ± 0.05 across all classes and time windows. The tCNN model performed best at detecting classes within 5-second windows, with AUROC >70%, specifically for “Tic” [0.79 (0.76-0.82)] and “Rest” [0.76 (0.73-0.79)].

Unlike tCNN, the average detection performance of 2-lCNN (0.60 ± 0.05) and transformer (0.55 ± 0.02) was subpar. The two models did not differ significantly from each other (*p* = 0.38) or at any time resolution (2-lCNN: *p* = 0.37; transformer: *p* = 1).

### Prediction Task

For class prediction (Table 3), tCNN (0.65 ± 0.05) and 2-lCNN (0.61 ± 0.08) outperformed the Transformer (0.56 ± 0.02) across all temporal windows and classes. Both models performed well above average within 1-second prediction windows across all classes (tCNN: (0.71 ± 0.05); 2-lCNN: (0.70 ± 0.03)), unlike the detection models, which showed coarser temporal performance. “Rest” (tCNN: ~0.71, 2-ICNN: ~0.72) and “Move” (tCNN: ~0.72, 2-ICNN: ~0.72) were the dominant classes predicted in the 1-second models. The average prediction AUROCs for the 5s and 10s windows were relatively low for both tCNN and 2-lCNN.

### Detection versus Prediction

We compared the models based on detection and prediction performance using average outcomes and the best window sizes (detection: 5s, prediction: 1s). Although the overall average tCNN detection AUROC (~66%) was higher than that of the best predictor models (tCNN: ~65%; 2-lCNN: ~61%) (Tables 2–3), there was no significant difference in AUROC between mean prediction and detection (p ≥ 0.05, left-tailed t-test) (Figure 2(a)). However, the best prediction window model (1s 2-lCNN) outperformed the best 5s 2-lCNN detection model (p=0.02), mainly because of its high voluntary movement and ‘rest’ AUROC (~72%) (Figure 2(b), Table 3). Nonetheless, “Tic” detection in longer 5-second windows remained dominant across all prediction and detection classes (AUROC (95% CI): 0.79 (0.76-0.82)), contrary to our study hypothesis (Table 2, Figure 2(c)). Overall, this suggests that models pre-trained on EMG labels and fine-tuned on ground-truth video labels based on expert opinion may perform better on tic-detection tasks than on prediction.

### Secondary Metrics

To address class imbalance, additional performance metrics, including the area under the precision–recall curve (AUPRC) and F1 Score, were evaluated (Table 4). Across all architectures, the mean AUPRC and F1-score values ranged from approximately 0.34 to 0.53 for detection and prediction tasks, indicating moderate classification performance under imbalanced conditions. Despite this limitation, all models demonstrated heightened sensitivity to 1-second temporal windows for both detection (tCNN: ~66%; 2-lCNN: ~65%; transformer: ~69%) and prediction tasks (tCNN: ~74%; 2-lCNN: ~71%) across all classes, highlighting the importance of short-duration EMG dynamics in tic-related activity. Secondary task-level metric performance, including positive predictive value (PPV) and negative predictive value (NPV), across each temporal window for all models, is detailed in the supplementary spreadsheet.

## Discussion

Our multimodal EMG–video models showed that detection of tic and non-tic states consistently outperformed prediction of future classes. Across all architectures, the best mean AUROC for detection was ~0.66 (tCNN), whereas prediction AUROCs were in the lower range (~0.55–0.64). In particular, the best tic-detection tCNN performance (AUROC ≈0.79) was on par with prior objective monitoring approaches. For example, wearable accelerometer-based detection achieved ~80% accuracy in one study ^19^, and a video-based deep CNN–LSTM achieved ~86% precision for tic movements in another study ^20^. Importantly, our results agree with recent findings: Barua et al. reported very high accuracy (≈97%) using simulated wireless signal data; however, such performance depended on non-clinical data ^21^. In contrast, our model was trained on clinical TS patient data, making it more representative of clinical scenarios.

Contrary to our hypothesis that short EMG windows would suffice, we found that longer EMG windows (5s) improved tic detection, likely due to better signal accumulation, noise smoothing, and the capture of the temporal structure of tics (Table 2). These findings mirror other biosignal studies where longer context improved accuracy; for example, cognitive-load detection was higher with longer windows ^22^. Conversely, predicting imminent tics favored the brief (1 s) window (Table 3), likely because only immediate pre-tic cues were informative in tic forecasting. These trends suggested that tics generated detectable signal patterns over several seconds and that temporal context improved detection. In summary, the precise timing of an upcoming tic relied on the most recent physiological activity.

Clinically, an automated system for detecting tics could vastly improve the monitoring and management of TS. Current methods rely on month-long retrospective scales (e.g., YGTSS) ^7^ or brief in-clinic videos ^8^. Both are subjective and limited in length ^23^. An AI pipeline combining EMG and video could enable continuous, objective monitoring outside the clinic setting. For example, caregivers could more objectively and remotely track tic frequency over days rather than relying on patient recall. These systems could also serve as endpoints in clinical trials by providing measurable data based on true changes in symptoms. Beyond monitoring, real-time tic detection may eventually be integrated into responsive therapies, such as closed-loop neurostimulation ^24^. Wearable devices are already exploring this possibility. For example, a recent prototype called “TSBand” uses wrist accelerometers, heart rate sensors, and other modalities to detect tic attacks and to alert caregivers ^25^. Our findings reinforce the potential of these approaches, demonstrating that multimodal machine learning can possibly improve tic detection.

However, our study had important limitations. Our dataset was relatively focused on subjects with implanted DBS systems under controlled conditions, which may have limited its generalizability. Our models primarily targeted motor tics, as surface EMG could not detect vocal tics or deep muscle activity; some facial tics may also have been missed because EMG sensors were not placed directly on the face. We relied on clinical expert video annotations as ground truth; however, inter-rater agreement was modest (IoU ~76%), reflecting the difficulty of labeling tics even for experts. This inter-rater and inter-subject variability in labeling could have led to an underestimation of any model’s true accuracy. The transformer model underperformed in this context, likely due to limited training and fine-tuning data for a large model. CNNs are generally better suited to capturing local temporal patterns ^26^ than transformers, and this represents a critical limitation of the study. Additionally, because the presence of any tic within a window resulted in a Tic label, longer windows had a greater opportunity to contain tic activity; this labeling strategy should therefore be considered when interpreting performance differences across window lengths. Future research should address these issues by collecting larger, more diverse EMG and video datasets (including recordings from real-world settings), incorporating additional modalities (such as audio for vocal tics), and exploring more advanced architectures once additional data is available.

In summary, our study demonstrated that CNN-based detection of tics in the setting of TS may possibly be more objective and convenient than traditional recall-focused clinical scales. By using realistic pretraining and multimodal inputs, our models achieved detection AUROCs approaching those of leading-edge models. These results support the further development of automated, AI-driven tic-monitoring tools for TS. Further improvement and validation of tools such as the one explored in the current study could enable continuous, quantitative assessment of tic severity, helping clinicians and researchers personalize and evaluate treatments for TS more effectively.

## Methods

### Subjects

Sixteen subjects (average age 27.3 ± 8.6 years), diagnosed with Tourette syndrome, agreed to participate in the study (Table 1) in accordance with the Declaration of Helsinki. Participants were recruited from the Norman Fixel Institute for Neurological Diseases as part of two related studies conducted from 2014 to 2025. They were excluded if they had: 1) simple motor tics, 2) predominant vocal tics, 3) psychotic symptoms, or 4) any other movement disorder besides TS. All participants had previously received deep-brain stimulation (DBS) implants as part of their clinical treatment. The studies received approval from the University of Florida Institutional Review Board (IRB202102161; IRB201300850) and are registered on clinicaltrials.gov (NCT02056873, NCT05371041).

### Recording Sessions

**Figure 1.**
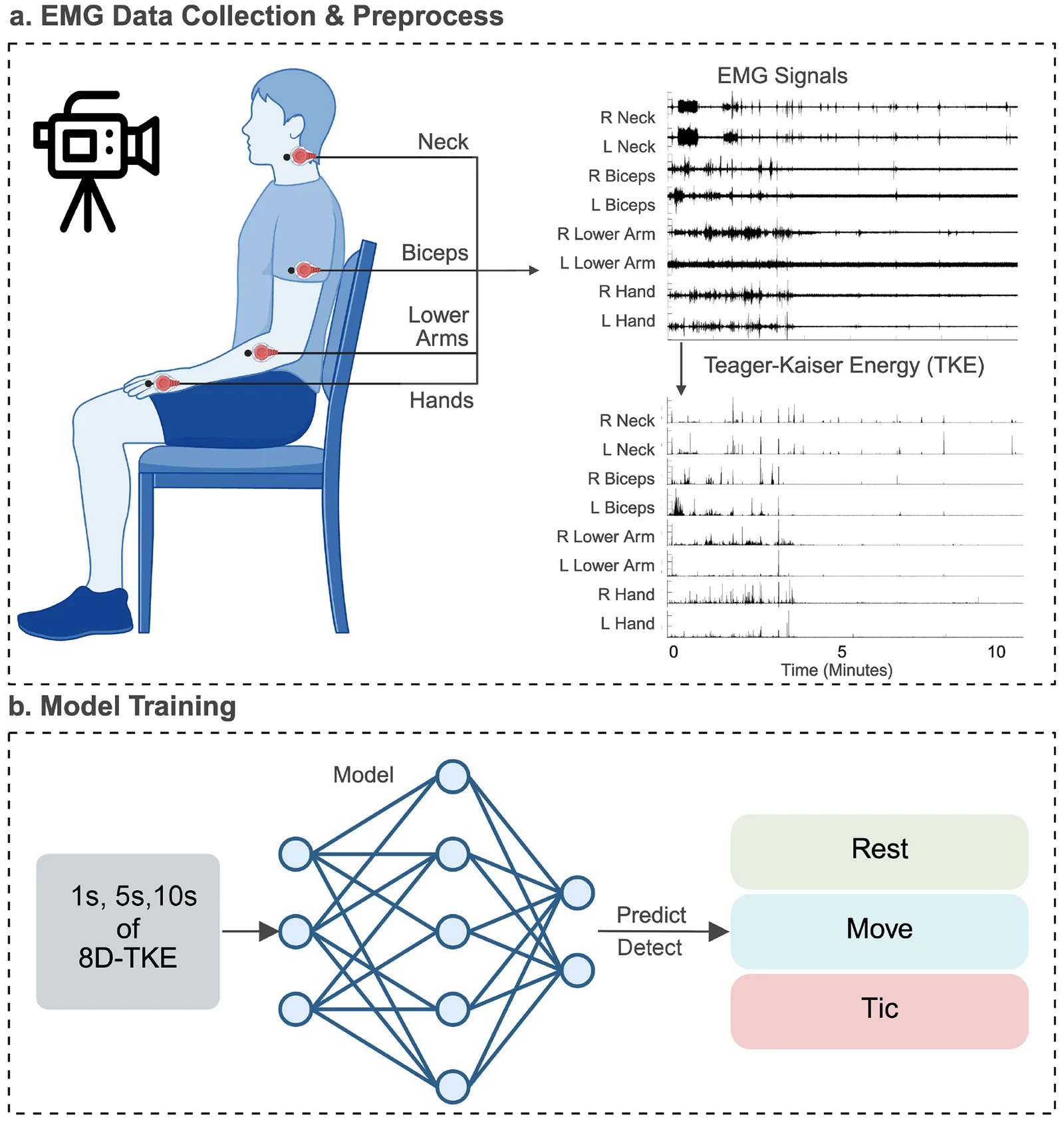
In the data collection stage, (**a**), we collected data from eight electromyography (EMG) sensors placed on the upper extremities and neck while concurrently recording video. Sensor numbering convention (even numbers for the right side (R) and odd numbers for the left (L)): 2-3 for sternocleidomastoid, 4-5 for biceps, 6-7 for flexor digitorum superficialis, 8-9 for extensor digitorum. Sensor 1 for EMG-to-video synchronization. EMG data were preprocessed to compute the 8D Teager-Kaiser energy (TKE) of the signal, which was used as input to the models. **(b)** The model received 1, 5, or 10-second windows of TKE features to predict or detect “rest,” “move,” or “tic.” The model architecture is detailed in the Supplementary Materials. *Abbreviations*: R, right; L, left

**Figure 2:**
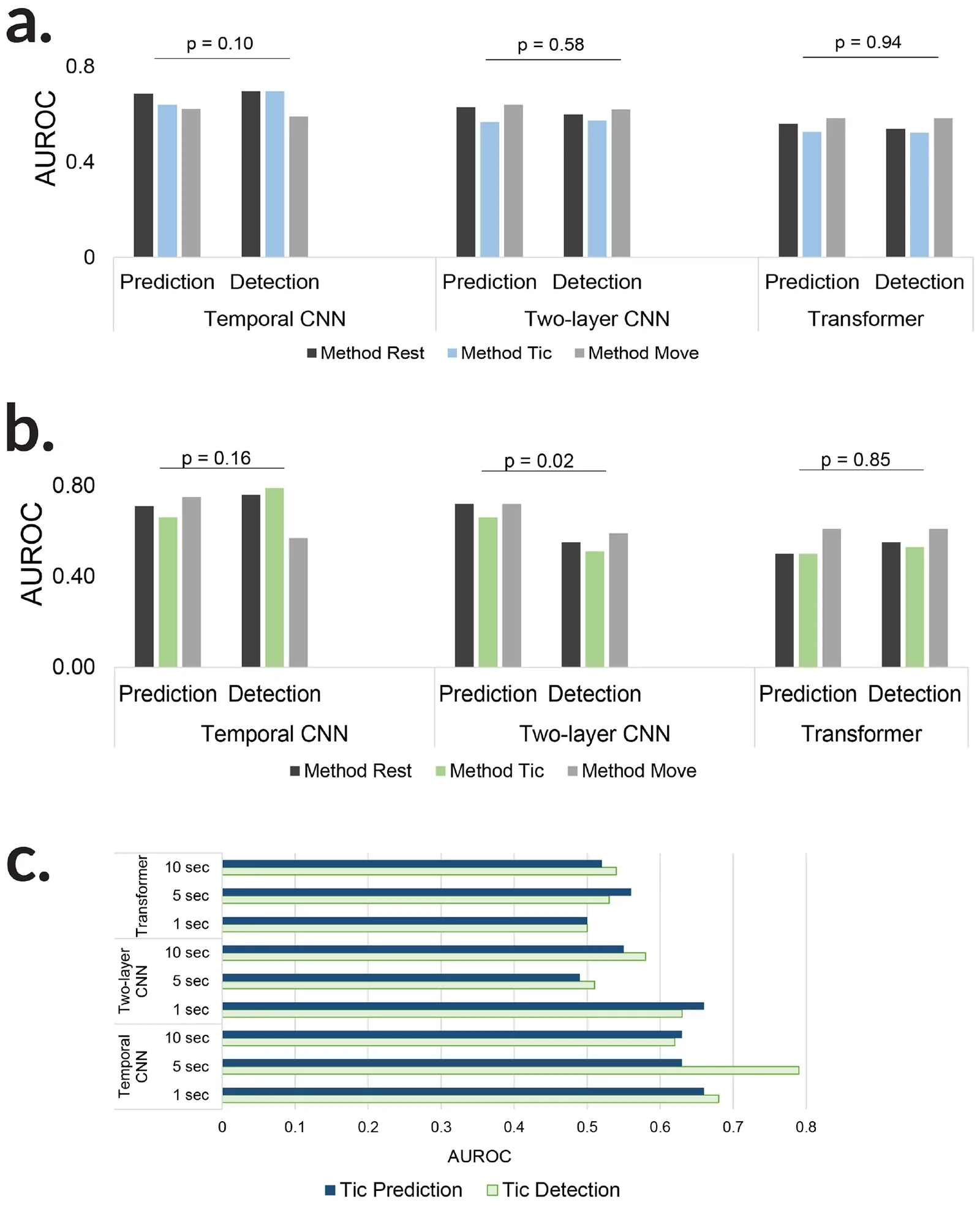
Prediction vs Detection Performance: **(a)** average outcomes across all time windows for rest (black), tic (blue), and voluntary movement (gray). There was no significant difference between model detection and prediction for all classes; **(b)**the best prediction models (in 1-sec windows) versus the best detection models (in 5-sec windows) for rest (dark green), tic (bright green), and voluntary movement (gray). There was no significant difference between prediction and detection outcomes across all classes, except for the two-layer CNN model, where prediction AUROCs greatly exceeded detection AUROCs. **(c)** Tic (TCNN) Detection (green) in 5-second windows outperformed tic prediction (dark blue). *Abbreviations:* AUROC, area under the receiver operating characteristic curve; CNN, convolutional neural network

Each subject’s participation was limited to at least 2 clinic visits per month for 3 consecutive months, resulting in 685 recording sessions (40.4 ± 28.4 recordings per subject; 6.7 ± 4.7 recordings per visit). Video data were captured using a front-facing Sony HD Handycam (1080p resolution, 30 frames per second, HDR-CX405; Sony, New York, NY, USA), and electromyography (EMG) signals were recorded from eightchannel Delsys Trigno Wireless sensors (1.1 kHz; Delsys Trigno system, Natick, MA, USA) placed on the upper extremities (Figure 1) ^12^. In each session, subjects were asked to rest, tic freely, and move voluntarily with DBS off to prevent stimulation artifacts in EMG recording ^27^. Video-to-EMG synchronization was achieved at the start of each recording session using the Florida Research Open-Source Toolbox (FROST) with the EMG base station, which activates a light-emitting diode (LED) in the camera’s view ^28^. Each session lasted an average of 15.3 (±2) minutes.

### Video Annotation

Four movement-disorder-trained experts independently reviewed full-session videos, marking tics and non-tic events. These expert raters were certified through the Collaborative Institutional Training Initiative program and, after IRB approval, were added to the IRBs as investigators before their involvement in research. Accordingly, informed consent was not obtained from the expert raters, who were part of the study team. Expert video annotations yielded an interrater reliability of 0.76 using Intersection over Union (IoU). The video recordings were uploaded to Label Studio (HumanSignal, online ^29^) to facilitate expert ground-truth annotation of tic and non-tic sequences using bounding boxes in frame-by-frame object tracking ^29^. Each annotated video had an average of 26,762 (± 17,421.97) frames (range, 330-123,330). Annotations were subsequently uploaded to a custom MATLAB (MathWorks, Cambridge, MA) tool to extract the marked events and their corresponding timestamps for synchronous EMG tracking. The average durations of the extracted video events were as follows: rest (10.29s ± 31.52), motor tic (2.17s ± 0.52), vocal tic (2.22s ± 0.89), left-hand (8.51s ± 6.06), and right-hand voluntary movements (8.68s ± 5.56) (Supplementary Fig.1). Only 141 videos were fully annotated due to labor costs associated with video-based tic evaluation systems ^23,30^. Tic annotations were restricted to tics that could be seen visually or heard audibly. Raters were blinded to the EMG data.

### EMG Data Preprocessing

Movement onset was detected in the EMG recordings using a double-threshold approach ^31^, similar to prior studies ^12,15^. The EMG signals were preprocessed using a zero-phase 6^th^-order Butterworth band-pass filter (1-450 Hz), followed by rectification and a zero-phase 4th-order Butterworth low-pass filter with a cutoff at 50 Hz. The double threshold entailed a Teager-Kaiser-computed amplitude threshold, *Th* and a temporal threshold, *Th*(*t*) to classify between rest (Rest), motor tic (Tic), and voluntary movement (Move) (Figure 1(a), Supplementary Fig. 2):

(1) Th=μ+j⋅σ(1)

(2) $$Th=\left\{\begin{array}{l} 1(\;\text{movement}\;)\\ 0(\;\text{no}\;\text{movement},\;\text{i.e.}\;\text{Rest}\;) \end{array}\right.$$

(3) $$h(t)=\left\{\begin{array}{ll} 1,(\;\text{Tic}\;),0.05s\leq t<2.67s & \\ 1,(\;\text{Move}\;) & t\geq 8.68s\\ 0,(\;\text{obsolete}\;) & \;\text{otherwise}\; \end{array}\right.$$

The amplitude threshold, *Th* was defined as a function of the mean (μ) and a scaled standard deviation (*j · σ*) of the signal’s Teager-Kaiser energy (TKE) ^32,33^ to detect the onset of movements: “Rest” and non-rest events (1,2). ‘*j’* was a tunable “resting” threshold parameter based on *σ* ensuring that *Th* was adapted to the background noise level of each EMG recording. To determine movement offset and to classify “Tic” from “Move” using a temporal threshold *Th(t)* at time *t*, the TKE envelope must exceed the amplitude threshold for at least 50 ms and up to ~2.7 seconds to qualify as a tic (3). Movements persisting beyond ~8.7 seconds were classified as voluntary (3). Once the envelope fell outside the defined thresholds, the EMG was marked as obsolete and discarded to preserve dataset integrity. The video-derived temporal threshold, *Th(t)* was determined based on the average event durations extracted from the corresponding video recordings. For tic presence and voluntary movement, the temporal threshold was limited to video durations of motor tics and right-hand movements, as EMG could only record muscle activity. Additionally, motor tic duration did not differ from vocal tic duration, nor did that of right-hand movements from left-hand movements (Supplementary Fig. 1).

Together, amplitude and video-derived temporal thresholds were used to refine the synthetic EMG training labels (n=544 recordings) and to inform model calibration.

### Model Training & Evaluation

The 8D-Teager-Kaiser energy (TKE) of the EMG signals from all eight channels served as the input to a temporal CNN (tCNN) ^34^, a 2-layer CNN (2-lCNN) ^26^, and a transformer ^35^ for delineating “Tic” and non-tic events (“Rest” and “Move”), as shown in Figure 1b. The tCNN used stacked dilated temporal convolutional blocks to capture temporal dependencies, whereas the 2-lCNN used two one-dimensional convolutional layers followed by pooling and fully connected classification layers. The Transformer was implemented as a study-specific sequence classifier based on the standard Transformer encoder architecture, with a 128-dimensional embedding, two encoder layers, four attention heads, and a 256-dimensional feed-forward layer. The final time-step representation was passed to three parallel classification heads to generate probabilities for “Rest”, “Tic”, and “Move.”

Our experiments evaluated the models on two tasks: detection and prediction. In the detection task, the models were trained to identify the label of the current observation window, classifying it as rest (Rest), motor tic (Tic), or voluntary movement (Move) based on the current 8D TKE feature segment. In the prediction task, the models were trained in a temporally predictive setting: given the current 8D TKE feature segment within 1-, 5-, and 10-second windows, the models predict the label of the subsequent time window, i.e., whether the next window corresponds to a tic or a non-tic, thereby enabling real-time anticipatory modeling of motor tics. Additional architectural and training details are provided in the Supplementary Materials to ensure reproducibility.

For window-level labeling, the sample-level annotations within each non-overlapping temporal window were aggregated using a priority-based rule. A window was labeled as Tic if a motor tic occurred at any time within the window. In the absence of a tic, a window was labeled as “Move” if any voluntary movement occurred. A window was labeled as “Rest” only if the entire window was free of voluntary or involuntary movement. When tic and voluntary movement annotations co-occurred within the same window, the Tic label was given priority. The same aggregation rule was applied to the subsequent window when constructing labels for the prediction task.

We used a fixed subject-level hold-out evaluation strategy. Subjects were randomly assigned to mutually exclusive training, validation, and test sets using a fixed random seed. The models were pretrained on EMG labels (n=476) and ground-truth video annotations (n=96) from nine subjects. Fine-tuning involved training on EMG labels (n=68) and ground-truth video annotations (n=25) from seven subjects. Validation (n=9) and testing (n=11) were conducted exclusively using ground-truth video labels from two randomly selected subjects (Table 1, Supplementary Table 1). No test-subject data were used in training or validation, nor were validation data used in training. Model selection was based on performance on the validation set, with the area under the receiver operating characteristic curve (AUROC) as the primary metric. The area under the precision–recall curve (AUPRC), sensitivity, and F1 score were reported as secondary metrics to address class imbalance. The best-performing model was then evaluated on the held-out test data.

Training was conducted on an NVIDIA L4 GPU using standard backpropagation with the Adam optimizer ^36^. Early stopping was applied based on validation AUROC to prevent overfitting. The input TKE features were normalized per channel.

### Statistical Analysis

Model performance metrics were compared using bootstrap-based ^37^ two-sample Welch and one-sample studentized *t*-tests with 10,000 bootstrap resamples at 95% confidence in R (version 4.6.1 ^38^). The analysis evaluated two-tailed differences in performance across model conditions while accounting for potential unequal variances and sample sizes. Where appropriate, directed (one-sided) hypotheses were tested with the “greater than” alternative to assess whether one model or temporal window significantly outperformed another. Statistical significance was assessed at *p* < 0.05.

## Supplementary Material

This is a list of supplementary files associated with this preprint. Click to download.

- supplementaryspreadsheet.xlsx
- Supplementarydatarevised.docx

## Figures and Tables

**Table 1:** Subject and Data Characteristics

Demography					

Number of participants, n	16				
Age (yr), mean (sd)	27.3 (8.6)				
Sex, female n (%)	12 (75)				
Disease duration, mean (sd)	18 (9.8)				
YGTSS, mean (sd)	83.5 (8.9)				
Detection Data	Pretrain		Finetune		

	Train	Val	Train	Val	Test
Number of participants, n	9	2	7	2	2
Number of recordings, n	572	9	93	9	11

Number of (10-s) samples, n	21265	709	1331	709	668
	
Rest, n (%)	6559 (30.8)	302 (42.6)	392 (29.5)	302 (42.6)	316 (47.3)
	
Move, n (%)	4661 (21.9)	105 (14.8)	146 (11.0)	105 (14.8)	115 (17.2)
	
Tic, n (%)	10045 (47.2)	302 (42.6)	793 (59.6)	302 (42.6)	237 (35.5)

Number of (5-s) samples, n	42030	1418	2591	1418	1383
	
Rest, n (%)	17738 (42.2)	802 (56.6)	1143 (44.1)	802 (56.6)	871 (63.0)
	
Move, n (%)	9038 (21.5)	180 (12.7)	263 (10.2)	180 (12.7)	195 (14.1)
	
Tic, n (%)	15254 (36.2)	436 (30.7)	1185 (45.7)	436 (30.7)	317 (22.9)

Number of (1-s) samples, n	208006	7454	13215	7454	7764
	
Rest, n (%)	129712 (62.4)	5496 (73.7)	8941 (67.7)	5496 (73.7)	6247 (80.5)
	
Move, n (%)	39265 (18.9)	731 (9.8)	1084 (8.2)	731 (9.8)	725 (9.3)
	
Tic, n (%)	39029 (18.8)	1227 (16.5)	3190 (24.1)	1227 (16.5)	792 (12.5)
Prediction Data	Pretrain		Finetune		

	Train	Val	Train	Val	Test
Number of participants, n	9	2	7	2	2
Number of recordings, n	572	9	93	9	11

Number of (10-s) samples, n	20790	702	1311	702	658
	
Rest, n (%)	6375 (30.7)	298 (42.5)	386 (29.4)	298 (42.5)	308 (46.8)
	
Move, n (%)	4517 (21.7)	105 (15.0)	148 (11.3)	105 (15.0)	115 (17.5)
	
Tic, n (%)	9898 (47.6)	299 (42.6)	777 (59.3)	299 (42.6)	235 (35.7)

Number of (5-s) samples, n	41555	1411	2570	1411	1374
	
Rest, n (%)	17422 (41.9)	797 (56.5)	1148 (44.7)	797 (56.5)	863 (62.8)
	
Move, n (%)	8929 (21.5)	180 (12.8)	256 (10.0)	180 (12.8)	195 (14.2)
	
Tic, n (%)	15204 (36.6)	434 (30.8)	1166 (45.4)	434 (30.8)	316 (23.0)

Number of (1-s) samples, n	207530	7447	13192	7447	7754
	
Rest, n (%)	129236 (62.3)	5490 (73.7)	8921 (67.6)	5490 (73.7)	6237 (80.4)
	
Move, n (%)	39265 (18.9)	731 (9.8)	1098 (8.3)	731 (9.8)	725 (9.4)
	
Tic, n (%)	39029 (18.8)	1226 (16.5)	3173 (24.1)	1226 (16.5)	792 (10.2)

Val, validation; sd, standard deviation; YGTSS, Yale Global Tic Severity Scale

**Table 2:** Detection Performance

Method	Window Size	AUROC (95% CI)			
		Detection			
		Rest	Tic	Move	mean (±sd)
Temporal CNN (tCNN)	1 sec	**0.67** (0.66-0.69)	**0.68** (0.65-0.70)	**0.63** (0.62-0.66)	0.66 (±0.03)
	5 sec	**0.76** (0.73-0.79)	**0.79** (0.76-0.82)	0.57 (0.53-0.61)	0.71 (±0.12)
	10 sec	**0.66** (0.62-0.71)	**0.62** (0.57-0.66)	0.57 (0.51-0.62)	0.62 (±0.05)

	mean (±sd)	0.70 (±0.06)	0.70 (±0.09)	0.59 (±0.03)	0.66 (±0.05)

Two-layer CNN (2-lCNN)	1 sec	**0.63** (0.61-0.64)	**0.63** (0.61-0.65)	**0.67** (0.65-0.69)	0.64 (±0.02)
	5 sec	0.55 (0.53-0.59)	0.51 (0.47-0.54)	0.59 (0.55-0.65)	0.55 (±0.04)
	10 sec	**0.62** (0.58-0.66)	0.58 (0.53-0.63)	**0.6** (0.55-0.66)	0.60 (±0.02)

	mean (±sd)	0.60 (±0.04)	0.57 (±0.06)	0.62 (±0.04)	0.60 (±0.05)

Transformer	1 sec	0.50 (0.50-0.50)	0.50 (0.49-0.52)	0.58 (0.56-0.59)	0.53 (±0.05)
	5 sec	0.55 (0.53-0.56)	0.53 (0.51-0.55)	**0.61** (0.58-0.65)	0.56 (±0.04)
	10 sec	0.57 (0.52-0.61)	0.54 (0.50-0.58)	0.56 (0.50-0.61)	0.56 (±0.02)

	mean (±sd)	0.54 (±0.04)	0.52 (±0.02)	0.58 (±0.03)	0.55 (±0.02)

Center values ≥ 0.6 are emphasized. AUROC, area under the receiver operating characteristic curve; CI, confidence interval; CNN, convolutional neural network; sd, standard deviation

**Table 3:** Prediction Performance

Method	Window Size	AUROC (95% CI)			
		Prediction			
		Rest	Tic	Move	mean (±sd)
Temporal CNN (tCNN)	1 sec	**0.71** (0.70-0.73)	**0.66** (0.65-0.68)	**0.75** (0.74-0.77)	0.71 (±0.05)
	5 sec	**0.68** (0.66-0.71)	**0.63** (0.60-0.66)	**0.62** (0.59-0.67)	0.64 (±0.03)
	10 sec	**0.67** (0.62-0.70)	**0.63** (0.58-0.67)	0.5 (0.43-0.57)	0.60 (±0.09)

	mean (±sd)	0.69 (±0.02)	0.64 (±0.02)	0.62 (±0.13)	0.65 (±0.05)

Two-layer CNN (2-lCNN)	1 sec	**0.72** (0.71-0.73)	**0.66** (0.63-0.68)	**0.72** (0.71-0.74)	0.70 (±0.03)
	5 sec	0.56 (0.53-0.58)	0.49 (0.46-0.52)	**0.63** (0.60-0.67)	0.56 (±0.07)
	10 sec	**0.61** (0.56-0.65)	0.55 (0.51-0.59)	0.57 (0.50-0.63)	0.58 (±0.03)

	mean (±sd)	0.63 (±0.08)	0.57 (±0.09)	0.64 (±0.08)	0.61 (±0.08)

Transformer	1 sec	0.5 (0.50-0.50)	0.5 (0.48-0.51)	**0.61** (0.59-0.63)	0.54 (±0.06)
	5 sec	0.59 (0.57-0.62)	0.56 (0.53-0.59)	0.57 (0.53-0.62)	0.57 (±0.02)
	10 sec	0.59 (0.55-0.62)	0.52 (0.47-0.54)	0.57 (0.50-0.61)	0.56 (±0.04)

	mean (±sd)	0.56 (±0.05)	0.53 (±0.03)	0.58 (±0.02)	0.56 (±0.02)

AUROC, area under the receiver operating characteristic curve; CI, confidence interval; CNN, convolutional neural network; sd, standard deviation

**Table 4:** Secondary metrics for Detection and Prediction Performance

Method	Window Size	Average 95% CI					
		Detection			Prediction		
		AUPRC	Sensitivity	F1-score	AUPRC	Sensitivity	F1-score
Temporal CNN (tCNN)	1 sec	0.41 (0.39-0.43)	**0.66** (0.62-0.70)	0.45 (0.43-0.47)	0.41 (0.39-0.42)	**0.74** (0.69-0.78)	0.46 (0.44-0.48)
	5 sec	0.51 (0.47-0.56)	**0.65** (0.58-0.73)	0.56 (0.53-0.59)	0.42 (0.39-0.46)	**0.68** (0.60-0.76)	0.49 (0.46-0.52)
	10 sec	0.44 (0.38-0.50)	0.55 (0.44-0.65)	0.54 (0.48-0.59)	0.41 (0.36-0.46)	0.59 (0.46-0.72)	**0.66** (0.60-0.71)

	mean	0.45 (0.41-0.49)	**0.62** (0.55-0.70)	0.52 (0.48-0.55)	0.41 (0.38-0.45)	**0.67** (0.58-0.75)	0.53 (0.50-0.57)

Two-layer CNN (2-lCNN)	1 sec	0.4 (0.38-0.42)	**0.65** (0.60-0.71)	0.44 (0.42-0.46)	0.41 (0.40-0.43)	**0.71** (0.66-0.76)	0.46 (0.44-0.47)
	5 sec	0.38 (0.34-0.42)	0.52 (0.42-0.63)	0.47 (0.42-0.52)	0.37 (0.32-0.42)	0.49 (0.39-0.59)	0.45 (0.38-0.53)
	10 sec	0.44 (0.37-0.50)	**0.62** (0.42-0.81)	0.52 (0.44-0.60)	0.4 (0.34-0.45)	0.51 (0.34-0.68)	0.46 (0.39-0.54)

	mean	0.40 (0.36-0.45)	**0.60** (0.48-0.72)	0.47 (0.43-0.52)	0.39 (0.35-0.43)	0.57 (0.46-0.68)	0.46 (0.40-0.51)

Transformer	1 sec	0.38 (0.35-0.41)	**0.69** (0.34-1.00)	0.43 (0.00-0.62)	0.4 (0.39-0.41)	0.18 (0.07-0.29)	0.13 (0.10-0.17)
	5 sec	0.42 (0.32-0.52)	0.55 (0.45-0.65)	0.4 (0.37-0.44)	0.36 (0.28-0.43)	0.52 (0.44-0.60)	0.47 (0.43-0.51)
	10 sec	0.36 (0.29-0.43)	0.57 (0.25-0.89)	0.44 (0.33-0.56)	0.33 (0.22-0.43)	0.46 (0.15-0.77)	0.42 (0.30-0.54)

	mean	0.39 (0.32-0.45)	**0.60** (0.35-0.86)	0.42 (0.31-0.54)	0.36 (0.30-0.43)	0.39 (0.22-0.55)	0.34 (0.28-0.40)

Average 95% CI values were calculated by taking the mean of the CI widths across class proportions, providing a summary that centers on the mean of the CI centers and standardizes precision across estimates. Center values ≥ 0.6 are emphasized. AUPRC, area under the precision-recall curve; CI, confidence interval; CNN, convolutional neural network

## Data Availability

The video and EMG data referenced in this paper will be uploaded to the NIH Data Archive for the BRAIN Initiative (DABI) upon study completion. The code used for model training and evaluation is uploaded to a GitHub repository ^39^: https://github.com/iheallab/multimodal-tic-detection
